# Classification of iliac vessels and selection of surgical approach and window in oblique lumbar interbody fusion at L5-S1 based on magnetic resonance imaging

**DOI:** 10.3389/fsurg.2026.1764671

**Published:** 2026-06-03

**Authors:** Zhao Liu, Kuan Lu, Fengyu Liu, Yuan Gao, Zhenfang Gu, Zhengqi Zhao, Xianze Sun

**Affiliations:** 1 Department of Spine Surgery, The Third Hospital of Shijiazhuang, Shijiazhuang, China; 2 Department of Orthopaedics, Hebei General Hospital, Shijiazhuang, China

**Keywords:** iliac vessels, L5-S1, MRI, oblique lumbar interbody fusion, surgical approach

## Abstract

**Objective:**

To investigate how the position of iliac vessels affects the choice of surgical approach (left or right oblique abdominal incision) and window (central or lateral) during L5-S1 oblique lumbar interbody fusion (OLIF), through imaging anatomy research.

**Methods:**

CT and axial T2-weighted MRI at L5-S1 were used to evaluate the iliac vessels’ position and classify the central vascular window (CW). Measurements were taken for the central window (CW), distance from the midline of the L5-S1 disc to the vessel's medial surface (DV), and psoas to iliac vein distances (LPV/RPV). Classification was based on the location of the common iliac vein/internal iliac artery: The intervertebral space was divided evenly from anterior to posterior into four zones—I, II, III, and IV. Based on iliac vein positions, patients were classified as follows: Type A (both veins in Zone I, left I/right I), Type B (left I/right II), Type C (left II/right I), Type D (both veins in Zone II, left II/right II), and Other Type (veins in Zone III or IV). A comparative analysis of imaging findings was performed.

**Results:**

302 patients were included (124 Type A, 30 Type B, 62 Type C, 82 Type D), and the remaining 4 patients, whose iliac veins were located in Zone III or IV, were defined as Other Type. Gender differences were observed, with Type A predominantly female. Type A had the smallest CW, while Type D had the largest. Type D showed the largest DV and RDV compared to other types. LPV and RPV were statistically significant between groups, with Type A and B having greater LPV than C and D.

**Conclusions:**

Classification based on iliac vessel positioning helps determine the optimal approach for OLIF: Left ATP-OLIF is preferred for Type A and B; Right ATP-OLIF or Left O-ALIF for Type C; Left O-ALIF for Type D. The classification simplifies approach selection, minimizing vascular and nerve damage.

## Introduction

The Oblique Lumbar Interbody Fusion (OLIF) technique, an innovative minimally invasive approach to lumbar fusion, was initially described by Silvestre and colleagues in 2012 (1). This method presents several advantages over traditional posterior lumbar interbody fusion (PLIF) or transforaminal lumbar interbody fusion (TLIF). Notably, OLIF spares the paraspinal muscles and ligaments, avoiding disruption to the spinal canal and nerve elements (2). Additionally, the utilization of a natural corridor situated between the psoas major muscle and the abdominal vasculature allows OLIF to mitigate the risk of lumbar plexus injury that may result from direct dissection of the psoas major muscle as seen in Extreme Lateral Interbody Fusion (XLIF). Furthermore, it minimizes potential harm to abdominal organs and anterior vertebral vessels, as well as damage to the superior hypogastric plexus, which are complications sometimes associated with traditional Anterior Lumbar Interbody Fusion (ALIF) (3). Consequently, the OLIF procedure has progressively become a favored surgical modality among spinal surgeons for addressing lumbar spinal pathologies.

In the initial stages of Oblique Lumbar Interbody Fusion (OLIF) surgery, the procedure is frequently utilized for L2-L5 interbody fusion (OLIF25). When addressing the L5/S1 segment, alternative approaches such as traditional Anterior Lumbar Interbody Fusion (ALIF) or posterior approaches often become necessary due to challenges associated with retraction of the iliac vessels and obstruction by the iliac crest. In cases involving multi-segmental lumbar pathologies, including L5-S1, it is common to first perform OLIF on the upper segments in a lateral decubitus position, followed by repositioning to access the L5-S1 segment, which significantly increases both surgical duration and anesthesia-related risks. In recent years, advancements in surgical techniques and improvements in related equipment have led to numerous clinical and basic studies on L5-S1 OLIF technology (4–7). The L5-S1 OLIF is essentially considered an ALIF performed in a lateral position through a retroperitoneal approach, utilizing an oblique lateral to medial trajectory relative to the iliac vessels to achieve discectomy and fusion. However, some scholars argue that the medial approach still poses a high risk of vascular and superior hypogastric plexus injury, proposing an alternative lateral iliac vessel approach to enter the intervertebral space (8, 9). To prevent confusion, Liu et al. (7) suggested a more precise nomenclature to distinguish between the two distinct surgical approaches for L5-S1 oblique lumbar interbody fusion. The medial approach to the iliac artery entering the intervertebral space is termed Oblique Anterior Lumbar Interbody Fusion (O-ALIF), whereas the lateral approach anterior to the psoas major muscle is referred to as Anterior to Psoas Oblique Lateral Interbody Fusion (ATP-OLIF). Both are derivatives of the L2-L5 oblique lumbar interbody fusion, representing anterior lumbar interbody fusion surgeries via oblique access. Additionally, some researchers advocate that right oblique abdominal incision approach may offer safer and more effective outcomes when addressing the L5-S1 intervertebral space (10). While there are reports on the vascular safety of the O-ALIF and ATP-OLIF approaches, consensus on the optimal surgical trajectory and whether a left or right-sided approach is safer remains elusive.

The objective of this investigation is to elucidate the relative positional relationships between blood vessels, intervertebral discs, and the psoas major muscle in the L5-S1 region through imaging studies employing MRI and CT. This aims to develop a novel vascular classification method that is straightforward, intuitive, and safe. The method should facilitate the reduction of intraoperative risks associated with iliac blood vessels, particularly the common iliac vein, and the superior hypogastric plexus. The findings are intended to provide a theoretical foundation and offer rational recommendations for selecting surgical strategies related to oblique lumbar interbody fusion at L5-S1, thereby contributing to improved patient outcomes.

## Method

The study was conducted in accordance with relevant guidelines and regulations, the ethical principles of the Declaration of Helsinki, and the Strengthening the Reporting of Observational Studies in Epidemiology (STROBE) guidelines. Ethical approval was obtained from the Ethics Review Committee of the Third Hospital of Shijiazhuang prior to the initiation of the study (Approval No.: 2023-062). Due to the retrospective nature of the study, written informed consent was waived by the Ethics Review Committee of the Third Hospital of Shijiazhuang. We conducted a retrospective analysis of imaging data (MRI and CT) from 435 patients who were admitted to our department for lumbar spine disease treatment between January 2022 and June 2022. The inclusion criteria were as follows: 1) age 18 years or older; 2) complete and clear lumbar MRI and CT imaging data. The exclusion criteria included: 1) presence of anatomical variations such as lumbarization or lumbar sacralization; 2) lumbar infections, tumors, fractures, or other pathological changes; 3) history of abdominal and/or lumbar spine surgery; 4) abdominal vascular abnormalities or diseases. Based on these criteria, a total of 302 patients were eligible for the study, comprising 140 males and 162 females, with ages ranging from 18 to 88 years (average age: 56.77 years). General patient data such as height, weight, and Body Mass Index (BMI) were collected.

**Figure 1:**
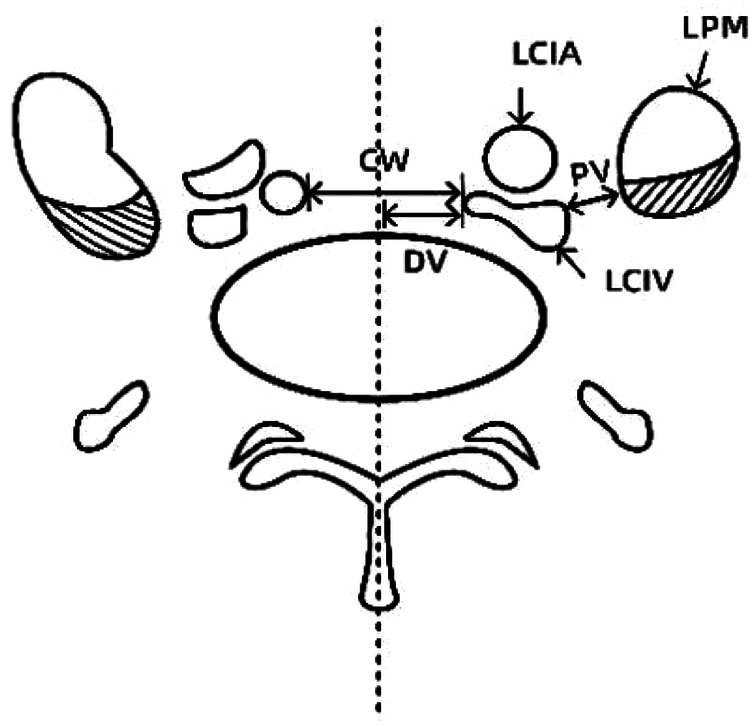
LPM: left psoas muscle. LCIV: Left common iliac vein. LCIA: Left common iliac artery. CW: the width of the central vascular window. DV: between the medial edge of the most medial vessels on both sides and the midline of the intervertebral disc. PV: between the lateral side of the common iliac vein/internal iliac vein and the medial side of the psoas.

All patients underwent 1.5T lumbar spine magnetic resonance imaging (MRI) and computed tomography (CT) with three-dimensional reconstruction examination. Lumbar MRI sagittal acquisition: slice thickness 4.0 mm, inter-slice gap 3.0 mm, echo time (TE) 120 ms, repetition time (TR) 2,175 ms, field of view (FOV) 239 mm × 239 mm, matrix size 264 × 185. Lumbar MRI axial acquisition: slice thickness 4.0 mm, inter-slice gap 3.0 mm, TE 110 ms, TR 2,500 ms, FOV 200 mm × 200 mm, matrix size 332 × 248. Lumbar CT sagittal acquisition: slice thickness 3–5 mm, inter-slice gap 3 mm. Lumbar CT axial acquisition: slice thickness 1–2 mm, inter-slice gap 1–3 mm. The PACS software was utilized for image review and data measurement. CT mid-sagittal and continuous scanning axial images were used to assess the location of the iliocava junction positions (JP). MRI axial images were employed to observe the types and vascular zones of the anterior vertebral vessels at the L5-S1 level, while also measuring the width of the central vascular window (CW), the distance between the medial edge of the most medial vessels on both sides and the midline of the intervertebral disc (DV), the distance between the lateral side of the common iliac vein/internal iliac vein and the medial side of the psoas major (PV), the presence of perivascular adipose tissue (PVAT), and any branches in the iliac vessels (Figure 1).

**Figure 2:**
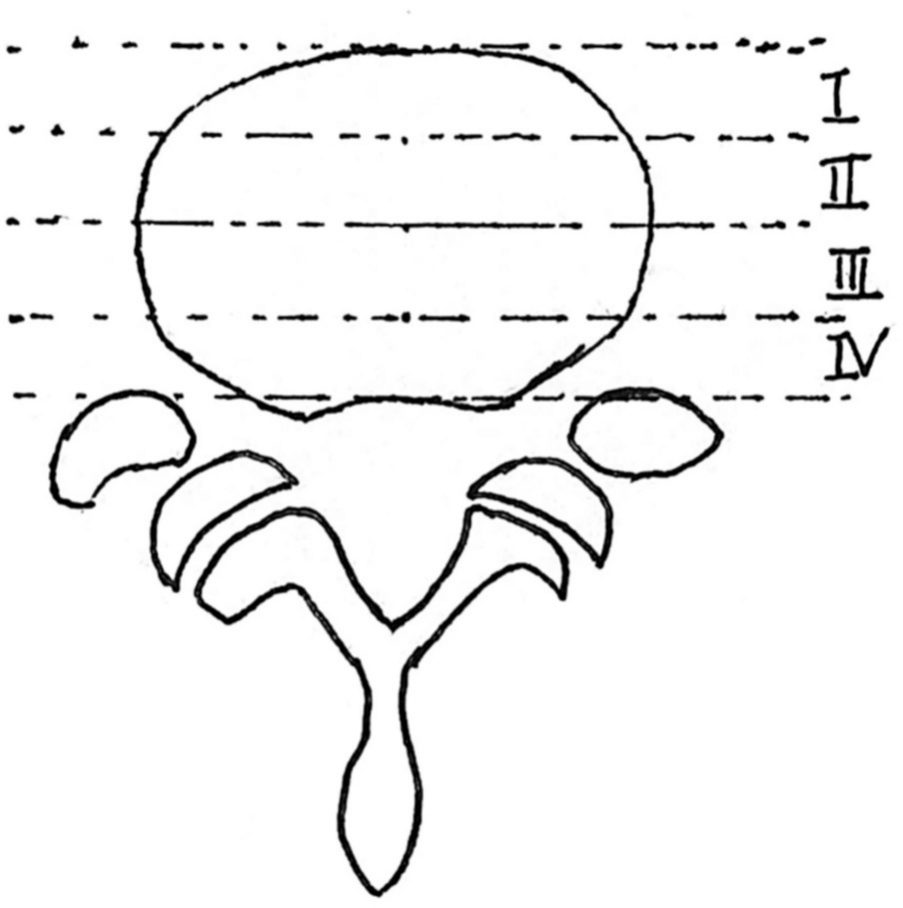
Vessel partitioning method: the disc space is divided into four zones equally from anterior to posterior: zones I, II, III and IV. The vessel division is defined according to the location of the posterior border of the prevertebral vessel, and if the posterior border of the vessel is on the demarcation line, the vessel is considered to be a posterior region, and if the posterior border of the vessel is located in the demarcation line between zone I and zone II, the vessel is judged to be zone II.

The specific methods are as follows:

1. Prevertebral Vascular Zoning: On T2-weighted MRI axial images at the mid-plane of the L5-S1 disc space, the intervertebral space is to be equally divided into four zones—Zone I, II, III, and IV (Figure 2)—from anterior to posterior, following the method proposed by Moro et al. (11). The vascular zone is determined based on the position of the posterior edge of the prespinal vessels. If the posterior edge of a vessel falls on a boundary line between zones, it is assigned to the posterior zone. For instance, if the posterior edge of a vessel is located on the boundary between Zone I and Zone II, the vessel is considered to be in Zone II. The location and zoning of the common iliac vein and artery on both sides should be observed and recorded. In cases where vessels have branched, the position of the internal iliac vein or artery should be measured.
2. Location of the Iliocava Junction Positions (JP): The location of the iliocava junction positions is determined using CT axial images. Measure the distance (Dj) from the inferior surface of the iliocava junction to the center of the L5/S1 intervertebral disc, as well as the distance (Dd) between the center of the L4/5 intervertebral disc and the center of the L5/S1 intervertebral disc. Subsequently, the junction position is calculated as a percentage of the total distance between these two points using the formula: JP = 100 − (Dj/Dd × 100). According to the classification method established by Capellades et al. (12), the JP is categorized into four groups: very high (JP values less than −33.3%), high (JP values between −33.3% and 33.3%), low (JP values between 33.4% and 66.6%), and very low (JP values greater than 66.7%).
3. Central Vascular Window (CW) and Vessel-Midline Distance (DV): Initially, on the L5/S1 MRI axial images, identify the type of vascular window. Classify the vascular window based on the type of the most medial vessels on the left and right sides into four types: artery-vein, artery-artery, vein-vein, or vein-artery. The width of the vascular window is defined as the distance between the inner side of the most medial vessel on the right and the inner side of the most medial vessel on the left. Furthermore, measure the distance from the inner margins of the most medial vessels on both sides to the midline of the intervertebral disc. If a vessel crosses the midline, this value is considered negative.
4. Vessel-Psoas Major Distance (PV): During the ATP-OLIF approach, the natural space between the iliac vessels and the psoas major muscle is typically utilized. Considering that the venous wall is thinner and less elastic than the arterial wall, making veins more susceptible to injury when retracted, we specifically measured the distance from the lateral margin of the common or internal iliac vein on both sides to the medial margin of the psoas major muscle.

All imaging data were measured in duplicate by a single independent spinal surgeon with a one-week interval between measurements, and the average of these two measurements was used as the final result. Regarding vascular zoning, three spinal surgeons performed two assessments with a one-week interval between them, and any discrepancies were resolved through discussion until a unanimous decision was reached. The Kappa value was calculated to estimate the reliability of both intra-observer and inter-observer conclusions.

### Statistical analysis

The statistical analysis was performed using the Statistical Package for Social Sciences (SPSS) version 21.0 (SPSS Inc., USA). All quantitative data underwent tests for variance homogeneity and normality. Continuous variables are presented as mean ± standard deviation. One-way analysis of variance (ANOVA) was used to analyze the differences in age, height, weight, body mass index (BMI), bilateral vascular psoas major space, central vascular window width, and the distance between the inner margins of the left and right vessels to the midline of the intervertebral disc among different vascular zones. The Chi-square test (*χ*^2^) or Fisher's exact probability method was used to analyze the differences in gender, common iliac vein confluence position, and the presence of perivascular fat among different vascular zones. The reliability and consistency of vascular position zoning were assessed through the degree of agreement within and between observers. Consistency was rated as fair, moderate, substantial, or excellent, with corresponding *κ* values of 0.21–0.4, 0.41–0.60, 0.61–0.8, and 0.81 and above, respectively. For all analyses, a *P* value <0.05 was considered statistically significant.

## Results

A total of 302 patients were included in this study, comprising 140 males and 162 females, with an average age of 56.77 years. General patient information, including height, weight, and body mass index (BMI), is presented in Table 1.

**Table 1:** General data of patients.

General information	Value
Age (years)	56.77 ± 13.92 (18–88)
Gender (M/F)	140/162
Height (m)	1.66 ± 0.07 (1.5–1.85)
Weight (Kg)	70.83 ± 11.25 (45–110)
BMI (Kg/m^2^)	25.63 ± 3.2 (17.8–33.51)

### Vascular zoning assessment

On the left side, the common iliac/internal iliac vein was located in zone I in 154 patients (51%), in zone II in 146 patients (48.3%), and in zone III in 2 patients (0.7%). On the right side, the common iliac/internal iliac vein was positioned in zone I in 186 patients (61.6%), in zone II in 114 cases (37.7%), and in zone III in 2 cases (0.7%). The left common iliac/internal iliac artery was found in zone I in 284 patients (94%), in zone II in 16 patients (5.3%), and in zone III in 2 patients (0.7%); on the right side, the common iliac/internal iliac artery was located in zone I in 290 patients (96%), and in zone II in 12 cases (4%).

**Figure 3:**
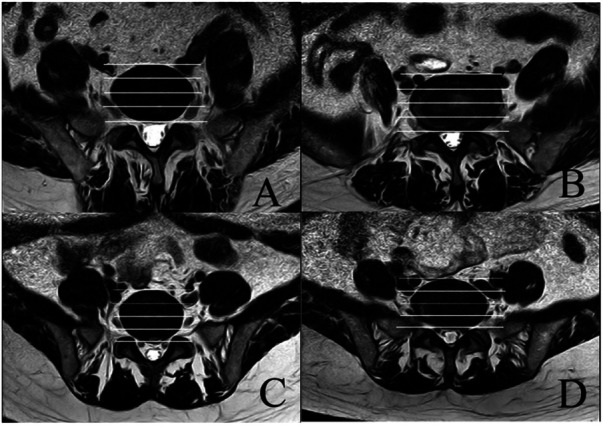
Division according to common/internal iliac vein location on both sides. Type **A**: left and right veins are located in zone I; type **B**: left veins are located in zone I and right veins are located in zone II; type **C**: left veins are located in zone II and right veins are located in zone I; type **D**: left and right veins are located in zone II.

The results indicated that the arteries on both the left and right sides were predominantly located in zone I, and since the venous wall is thinner and more susceptible to injury during surgery, we classified the types based on different venous zones on the left and right sides. Given that a very small number of patients had veins located in zone III (two patients each side), they were excluded from the classification study. Therefore, the final classification method and results are as follows: Type A, where both left and right veins are located in zone I; Type B, where the left vein is in zone I and the right vein is in zone II; Type C, where the left vein is in zone II and the right vein is in zone I; Type D, where both left and right veins are located in zone II (Figure 3). Out of the total 298 patients included in the study, there were 124 patients with Type A, 30 with Type B, 62 with Type C, and 82 with Type D.

Table 2 provides a detailed record of the general information and anatomical parameter results and differences among different classifications. There were no statistically significant differences in age, height, weight, and BMI (*P* values were 0.186, 0.506, 0.208, and 0.366, respectively).

**Table 2:** Comparison of general data and anatomical parameters of different vascular location types.

General information	Type A	Type B	Type C	Type D	*P* value
Number of patients (n)	124	30	62	82	-
Age (years)	59.06 ± 15.93	60.67 ± 13.78	58.64 ± 13.49	57.65 ± 14.53	0.186
Gender (M/F)	52/72	18/12	30/32	58/24*	0.001
Height (m)	1.68 ± 0.76	1.64 ± 0.72	1.67 ± 0.8	1.64 ± 0.75	0.506
Weight (Kg)	70.46 ± 9.25	71.83 ± 9.04	69.5 ± 15.53	68.38 ± 7.9	0.208
BMI (Kg/m^2^)	26.21 ± 2.82	26.71 ± 3.61	24.92 ± 3.15	25.84 ± 2.18	0.366
Width of central vascular window (mm)	32.34 ± 10.57	35.03 ± 10.67*	36.34 ± 6.11*	43.76 ± 7.31*^,^^#^^,^^&^	0.000
Distance from medial side of vessel to midline of intervertebral disc (mm)					
LEFT	14.71 ± 8.54	14.84 ± 8.39	19.81 ± 4.93*^,^^#^	22.67 ± 4.99*^,^^#^^,^^&^	0.000
Right	17.58 ± 4.46^†^	20.19 ± 3.86*^,^^†^	16.52 ± 5.14^#^^,^^†^	21.09 ± 3.61*^,^^&^^,^^†^	0.005
Vascular psoas muscle distance (mm)					
LEFT	4.94 ± 3.53	5.56 ± 2.79	1.58 ± 0.81*^,^^#^	1.23 ± 0.78*^,^^#^	0.000
Righ	2.53 ± 1.65^†^	1.13 ± 1.17*^,^^†^	2.62 ± 0.91^#^	1.15 ± 0.36*^,^^&^	0.029
Extravenous fat layer (n absent/present)					
LEFT	56/68	14/16	22/40	42/40	0.31
Righ	24/100^†^	12/18^†^	14/48^†^	13/54^†^	0.095

* Indicates a statistically significant difference compared with Type A.

# Indicates a statistically significant difference compared with Type B.

& Indicates a statistically significant difference compared with Type C.

† Indicates a statistically significant difference between the left and right sides within the same type.

The distribution of central vascular window types showed no statistical difference among the four types (*P* = 0.206).

Gender differences were statistically significant across the four types (*P* = 0.001), where a higher proportion of female patients were of Type A compared to Type D, and a higher proportion of male patients were of Type D compared to Type A.

The central vascular window widths for patients of Types A, B, C, and D were: 32.34 ± 10.57 mm, 35.03 ± 10.67 mm, 36.34 ± 6.11 mm, and 43.76 ± 7.31 mm, respectively, showing statistically significant differences. Type D was significantly larger than Types A, B, and C, with Types B and C being larger than Type A (all *P* values <0.05).

Statistically significant differences were found in the comparison of bilateral vascular-midline distances (left side *P* = 0.000, right side *P* = 0.005). In intergroup comparisons, patients with Type D had the greatest left vascular-midline distance, whereas those with Type A had the smallest. Patients with Types B and D had significantly greater right vascular-midline distances compared to Types A and C. In intragroup comparisons, Types A, C, and D showed a significantly greater right vascular-midline distance than the left, while patients with Type B had a significantly greater left vascular-midline distance than the right (all *P* values <0.05).

Statistically significant differences were observed in the distance between the bilateral vessels and the psoas major muscle (left side *P* = 0.000, right side *P* = 0.029). In intergroup comparisons, patients with Types A and B had a significantly greater left vessel-psoas major distance than those with Types C and D, while patients with Types A and C had a significantly greater right vessel-psoas major distance than those with Types B and D (all *P* values <0.05). In intragroup comparisons, patients with Types A and B showed a significantly smaller right vessel-psoas major distance compared to the left (all *P* values <0.05), whereas no significant differences were found between the left and right sides for patients with Types C and D (all *P* values >0.05).

The presence of PVAT on the left and right sides showed no significant differences among the four types (*P* values were 0.31 and 0.095, respectively). However, within-group comparisons revealed that the proportion of patients with PVAT on the right side was significantly higher than on the left for all types (all *P* values <0.05). The average age of patients with PVAT on the left was 55.16 ± 13.36 years, while for those without it, the average age was 58.81 ± 14.55 years, showing a statistically significant difference (*P* = 0.025); on the right side, the average age of patients with PVAT was 55.59 ± 14.03 years, and for those without it, the average age was 60.21 ± 13.45 years, which also showed a statistically significant difference (*P* = 0.012). The results indicate that age is related to the presence of PVAT; The study indicates that the presence of PVAT decreases with increasing age. Among patients with PVAT on the left, there were 70 males and 94 females, while among those without it, there were 70 males and 68 females, showing no statistical difference in gender (*P* = 0.163); On the right side, patients with PVAT included 100 males and 120 females, and those without it included 40 males and 42 females, with no statistical difference in gender (*P* = 0.606), indicating that gender is not related to the presence of PVAT.

There was a statistically significant difference in the iliocava junction positions among the four patient types, with the number of patients with different iliocava junction positions for each type shown in Table 3. Types A and D exhibited significant differences in the various iliocava junction positions, including extremely high, high, low, and extremely low (all *P* values = 0.000). Notably, patients with an extremely low iliocava junction positions were only observed in Type A. The proportion of Type A patients with a low or extremely low iliocava junction positions was significantly higher than in Type D, while the proportion of Type D patients with a high or extremely high iliocava junction positions was significantly higher than in Type A.

**Table 3:** Comparison of different vessel location types and iliac vein confluence.

Iliocaval junction positions	Group A	Group B	Group C	Group D
Very high	10	4	10	14*
High	60	18	38	58*
Low	42	8	14	10*
Very low	12	0*	0*	0*

This table shows the number of patients with different iliocaval junction positions across the four venous classification types.

*Indicates a statistically significant difference in iliocaval junction position compared with Type A. The comparison refers to the four categories of iliocaval junction positions (very high, high, low, very low).

The intra-observer reliability analysis for the measurements of the central vascular window width, vascular-disc midline distance, and vascular-psoas major distance showed *κ* values of 0.94, 0.94, and 0.93, respectively, representing excellent reliability. The intra-observer and inter-observer consistency analyses for vascular position zoning are presented in Tables 4, 5. The internal reliability among three observers was excellent (*κ* values of 0.95, 0.92, and 0.94, respectively), indicating a strong intra-observer consistency in the zoning results; the inter-observer reliability between observer 1 and 2, observer 1 and 3, and observer 2 and 3 was also excellent (*κ* values of 0.90, 0.86, and 0.88, respectively), indicating a strong inter-observer consistency in the zoning results.

**Table 4:** Agreement of vessel location zonation observed 2 times interval between 3 observers.

Observer	Number of subjects with consistent classification	Percent of Typing Agreement (%)	Kappa Coefficient
1	290	97.3	0.95
2	285	95.7	0.92
3	288	96.8	0.94

**Table 5:** Agreement of vessel location partitioning between 3 observers.

Observer	Number of subjects with consistent classification	Percent of Typing Agreement (%)	Kappa Coefficient
1, 2	282	94.6	0.90
1, 3	274	91.9	0.86
2, 3	277	93.1	0.88

## Discussion

The Oblique Lumbar Interbody Fusion (OLIF) approach for the treatment of lumbar spinal diseases was first reported by Mayer (13) in 1997. Subsequently, Silvestre (1) and colleagues coined the term OLIF in 2012 as the nomenclature for the retroperitoneal anterolateral lumbar fusion procedure. The OLIF technique utilizes the natural space between the abdominal vessels and the left psoas major muscle to access the intervertebral disc. Compared to traditional Anterior Lumbar Interbody Fusion (ALIF), it does not require the dissection and retraction of major abdominal vessels, significantly reducing the risk of complications such as abdominal organ injury, vascular damage, retrograde ejaculation, and intestinal adhesions (14–17). Additionally, because it avoids traversing the psoas major muscle, it effectively prevents complications such as lumbar plexus injury that can be caused by extreme lateral interbody fusion (XLIF) (18). Numerous studies and reports on OLIF have been published to date, demonstrating its favorable clinical outcomes, short operative time, minimal blood loss, rapid recovery, and high fusion rates (3, 8, 19). As a result, OLIF has gained increasing favor among scholars both domestically and internationally.

OLIF was generally used to fuse the disc space between L2 and L5 (OLIF25) at the beginning of development. Due to the influence of the iliac crest and greater variability in prevertebral blood vessels, there is a higher risk of injury at the L5/S1 level. Many surgeons prefer using an traditional Anterior Lumbar Interbody Fusion (ALIF) or posterior approach for this area. In a study by Molinares et al. (20), found that only 69% of lumbar MRI images had an oblique corridor of access to the L5/S1 disc. Results from Silvestre et al. (1) suggest that OLIF surgeries are safe for all segments from L2 to L5, but the risk of damaging iliac vessels during OLIF surgery at L5/S1 is significant, leading to the recommendation for ALIF procedures. In such cases, when dealing with multi-segmental lumbar spinal pathologies including L5/S1, it is often necessary to complete the upper segment OLIF surgery in a lateral decubitus position, then change the surgical position for the L5/S1 ALIF surgery in a supine position or PLIF/TLIF surgery in a prone position. An increase in surgery time means higher risks associated with both the surgery and anesthesia.

In recent years, with the continuous improvement of surgical techniques and the constant upgrading of surgical equipment and instruments, oblique lumbar interbody fusion at L5-S1 (OLIF51) has gradually become a hot research topic in the field of spinal surgery. Woods et al. (3) performed OLIF surgeries on 340 discs from 137 patients, including OLIF25, OLIF51, and a combination of OLIF25 with OLIF51. They detailed the surgical technique for OLIF51, adopting an approach medial to the iliac vessels (O-ALIF), and concluded that OLIF is a safe procedure applicable to both L2-5 and L5-S1 levels. Berry et al. (4) performed OLIF surgeries on 87 patients with lumbar spine diseases, also using the medial approach to the iliac vessels for L5/S1 OLIF (O-ALIF). Their results showed that this surgical method is safe and feasible, and that addressing L5/S1 and upper segments simultaneously with OLIF does not increase early complications. Compared to traditional ALIF, the advantage of L5-S1 OLIF (O-ALIF) is that it avoids damaging the rectus abdominis muscle and its innervating nerves, causes minimal traction on abdominal organs, and is suitable for obese patients. Meanwhile, L5-S1 OLIF (O-ALIF) can complete multilevel lumbar fusion including L5-S1 in the same position and is indicated for obese patients.

The oblique lumbar interbody fusion at the L5-S1 level is commonly considered a variation of anterior lumbar interbody fusion (ALIF) approached from an oblique trajectory through a retroperitoneal route, known as O-ALIF. Typically, this procedure is performed with the patient in the right lateral decubitus position, incised from the left side of the abdomen, accessing the L5-S1 disc space through the bifurcation of the iliac vessels (3, 5, 21). However, it has been suggested that it is safer to perform O-ALIF at L5-S1 through a right abdominal incision in the left lateral decubitus position. Song et al. collected magnetic resonance imaging (MRI) data from 274 patients to compare the anatomical characteristics of blood vessels at the L5-S1 level on both the left and right sides. Their results showed that in 76.3% of patients, the left-sided vessels were located medial or central, while in 90.5% of patients, the right-sided vessels were located central or lateral. The more medial the vessel position, the more it obscures the central surgical window. Additionally, the authors also found that the incidence of perivascular adipose tissue around the right vessels at the level of the L5-S1 intervertebral space was significantly higher than that on the left, indicating that the right vessels were easier to pull than the left vessels. Therefore, the authors conclude that approaching L5-S1 O-ALIF surgery from the right side is safe and reliable. Despite the increasing maturity of the L5-S1 OLIF (O-ALIF) technique, some scholars believe that the intervascular approach can sometimes be obstructed by iliac vessels, significantly increasing the difficulty and risk of surgery (16, 22). Also, due to the location of the superior hypogastric plexus anterior to the L5-S1 interval, intraoperative damage leading to retrograde ejaculation in males is a concern that warrants close attention (23). Hence, an alternative surgical protocol (ATP-OLIF) via anterior psoas muscle and lateral iliac vessel approach has been proposed, which can effectively reduce the injury of the anterior vessels of the intervertebral space and the superior hypogastric plexus. Zairi et al. (8) performed OLIF surgery on six patients with multisegmental lumbar disease, including L5-S1, using the left abdominal incisional ATP-OLIF technique, and the results showed this method to be safe and feasible. Miscusi (24) performed left-side ATP-OLIF surgery on 32 patients with degenerative L5-S1 disease or those undergoing revision after posterior surgery, achieving satisfactory clinical and radiological outcomes. Berry et al. (4) used three different approaches for the L5-S1 OLIF procedure, 1) a left-sided intrabifurcation approach;2) left-sided prepsoas approach; 3) right-sided prepsoas approach. The results indicated that all three approaches for L5-S1 OLIF surgery were safe and reasonable.

In order to reduce the risk of iliac vessel injury during surgery, a large number of studies on the anatomy and imaging of the iliac veins have emerged, most of which focus on the impact of vessel position, morphology, and classification on the safety of O-ALIF surgery (11, 25). However, there are fewer studies on the vessel position and classification related to ATP-OLIF. Liu et al. (7) classified the left common iliac vein on coronal images based on the position of the medial edge of the vein and the presence or absence of surrounding adipose tissue and adhesion to the intervertebral space into types I, II, and III. On sagittal images, they classified the left common iliac vein based on the position of the posterior edge of the vein and the presence or absence of surrounding adipose tissue and adhesion to the intervertebral space into types A, B, and C. They recommended that patients with types I and II are suitable for O-ALIF, while those with types A and B are suitable for ATP-OLIF. Zhang et al. (26) introduced the “V-line” as a basis for different approaches to L5-S1 OLIF. They suggested that if most of the iliac vein is located ventral to the V-line, L5-S1ATP-OLIF should be performed, while if most of the iliac vein is located dorsal to the V-line, the O-ALIF approach should be used. The above studies on vessel classification for surgical approach selection are limited to the left-side approach, and currently, there are no imaging studies that can simultaneously guide the choice of left or right side and whether to choose O-ALIF or ATP-OLIF.

The objective of this study is to propose a novel, simple, intuitive, and safe vascular position classification method based on magnetic resonance imaging (MRI) and computed tomography (CT) studies of the local vascular structure at the L5-S1 level. This method aims to provide a basis and reference for selecting surgical strategies in L5-S1 oblique lumbar interbody fusion. The study selects T2-MRI axial images of the middle plane of the L5-S1 intervertebral space and divides the intervertebral space into four equal zones from anterior to posterior: Zones I, II, III, and IV. The vascular zones are defined according to the posterior edge position of the prevertebral vessels. The advantage of this zoning system is that it intuitively reflects the relative positional relationship between the left and right sides of the vessels, the psoas major muscle, and the intervertebral disc. Particularly when selecting an external iliac vessel approach, it can directly show whether the vessels obstruct the surgical window. Our study results show that in 99.3% of patients, the veins on both sides are located in Zones I and II, with 0.7% of veins positioned in Zone III.。。The arteries on both sides are predominantly located in Zone I, with 94% on the left side and 96% on the right side. Considering that veins have thinner walls and are more frequently adhered to the intervertebral disc, they are more susceptible to damage during surgery. Therefore, we have classified the positions based on the different locations of the bilateral veins, which may be more instructive: Type A, where both left and right veins are located in Zone I; Type B, where the left vein is in Zone I and the right vein is in Zone II; Type C, where the left vein is in Zone II and the right vein is in Zone I; Type D, where both left and right veins are located in Zone II. For Type A patients, the width of the central vascular window is the smallest, measuring 32.34 ± 10.57 mm (range 6.92–45.36 mm), indicating a greater difficulty and higher risk when approaching through the central vascular window. This may be related to a low confluence position of the iliac veins and a larger proportion of patients with extremely low positions (accounting for 43.55%). Molinares et al. (20) suggest that the iliocava junction positions is closely related to the possibility of entering the L5-S1 disc space; the lower the iliocava junction positions, the smaller the likelihood of finding a ventral channel to the L5-S1 disc space, which is consistent with our study results. Intragroup comparison shows that the distance between the left iliac vein and the psoas major muscle is significantly greater than on the right side (4.94 ± 3.53 vs. 2.53 ± 1.65 mm, *P* < 0.05), which may be related to the right vein being closer to the dorsal side compared to the left (27). Therefore, for Type A patients, it is recommended to preferentially choose the left ATP-OLIF approach because the common iliac vein is located in front of the surgical window (Zones II and III), eliminating the need for retraction or ligation and division of the iliolumbar veins, and avoiding injury to the superior hypogastric plexus below the vascular bifurcation. For B-type patients, the central vascular window width is 35.03 ± 10.67 mm, with the distance from the left side of the blood vessel to the intervertebral disc midline being significantly less than that on the right side (14.84 ± 8.39 vs. 20.19 ± 3.86 mm, *P* < 0.05). Additionally, the distance between the left side blood vessel and the psoas major muscle is significantly greater than that on the right side (5.56 ± 2.79 vs. 1.13 ± 1.17 mm, *P* < 0.05). We suggest that for B-type patients, the left ATP-OLIF approach should be chosen first. This is because the left iliac vein is located in Zone I, regardless of whether the vein is adhesive or not, the lateral approach window of the left iliac vessels is large enough. Furthermore, the left approach is suitable for single or multiple segments, making the choice of the left ATP-OLIF approach safe. For C-type patients, the central vascular window width is 36.34 ± 6.11 mm, with the distance from the medial side of the left blood vessel to the intervertebral disc midline being significantly greater than that on the right side (19.81 ± 4.93 vs. 16.52 ± 5.14 mm, *P* < 0.05). Although there is no significant difference in the distance between the blood vessels and the psoas major muscle on both sides, the left iliac vein is located more posteriorly in Zone II, which may more likely obstruct the lateral surgical window, making the right iliac vascular lateral approach safer and more reliable. Therefore, for C-type patients, we recommend that patients undergoing single-segment L5-S1 surgery should prefer the right ATP-OLIF approach to avoid retrograde ejaculation caused by injury to the superior hypogastric plexus during surgery. For young male patients requiring multi-segmental disc treatment, since younger patients have milder intervertebral disc degeneration, the likelihood of adhesion of the left iliac vein is lower, and the upper disc requires a left-sided approach, a left ATP-OLIF approach can be used. If the surgery involves multi-segmental discs in female patients or elderly male patients, a left O-ALIF approach is recommended. For D-type patients, the central vascular window width is the largest, averaging 43.76 ± 7.31 mm, while the gap between the blood vessels and the psoas major muscle is the smallest on both sides. Therefore, choosing an intervascular approach is safer. Intragroup comparisons show that the distance from the medial side of the left blood vessel to the midline of the intervertebral disc is significantly greater than that on the right side (22.67 ± 4.99 vs. 21.09 ± 3.61 mm, *P* < 0.05). Hence, for D-type patients, we suggest that female or elderly male patients should prefer the left O-ALIF approach, whereas for single-segment young male patients, the ATP-OLIF approach on the non-adhesive side can be chosen based on whether the iliac vein has adhesions or not. If dealing with multi-segmental spaces in young male patients, since the possibility of adhesion of the left iliac vein is lower, the left ATP-OLIF can be performed.

For the ATP-OLIF approach, when the iliac vessels are located in Zone I, there is generally no need to consider adhesion between the blood vessels and the intervertebral disc, nor is there a need to consider ligation of the iliac-lumbar vein during surgery. However, when the iliac vessels are located in Zone II, it is essential to consider whether there is adhesion between the vessels and the intervertebral space, and ligation of the iliac-lumbar vein must be considered during surgery. In this study, for Types B, C, and D, there is one side where the common iliac vein or internal iliac vein is located in Zone II. If choosing to perform ATP-OLIF on the side where the common iliac vein is located in Zone II, it is crucial to carefully observe the adhesion of the common iliac vein and the condition of the iliac-lumbar vein. Only by ligating and cutting the iliac-lumbar vein and retracting the common iliac vein anteriorly can an ideal surgical window be exposed (7).

We have conducted a consistency evaluation of the method for classifying the position of the iliac vessels. The results show that the reliability within observers and between observers is excellent, indicating that the classification results have strong intra-observer consistency. We have conducted a consistency evaluation of the method for classifying the position of the iliac vessels. The results show that the reliability within observers and between observers is excellent, indicating that the classification results have strong intra-observer consistency.

This study has several limitations. First, there may be some degree of human error in measuring each parameter. Second, the subjects are relatively homogeneous and do not include special cases such as lumbar deformity, sacralization/lumbarization of the sacrum. Third, this study is based on imaging measurements from CT and MRI scans in the supine position, and it does not analyze whether the imaging parameters of the iliac vessels change in different body positions. Fourth, our determination of the location of the iliac vein confluence is made by combining axial CT with mid-sagittal images. However, in axial images, it is sometimes difficult to see the exact location of the confluence, and it is almost impossible to determine the mode of venous confluence and the angle at the confluence. Additionally, our hospital's MRI examinations often do not include coronal views, and results might be more accurate if coronal lumbar MRI and corresponding regional MRA examinations were used. Fifth, the results of this anatomical imaging study should be combined with clinical findings to further validate our conclusions, which will be our next research goal. Sixth, this study excluded anatomical variations such as lumbarization or sacralization, which may affect vascular anatomy and thus limit the applicability of this classification method to a broader population. Patients classified as Other Type (veins in Zone III or IV) were few in number and had a large central vascular window, making them directly suitable for O-ALIF; however, compared with the other four types, the applicability of the classification to these patients is more limited. Seventh, although all quantitative measurements (CW, DV, PV) were repeated by a single observer with excellent intra-observer reliability (*κ* = 0.93–0.95), the absence of multiple independent observers may introduce bias.

## Conclusion

The classification is based on the position of the common/internal iliac veins bilaterally in zones I-IV, categorized into Type A, B, C, and D. For Type A and B, ATP-OLIF with left oblique abdominal incision is the preferred approach. For Type C, the right-sided ATP-OLIF is favored for single-segment cases, while for multi-segment cases, the choice between left ATP-OLIF and left O-ALIF is determined by the patient's gender, the presence of a fatty gap between the left common/internal iliac vein and the intervertebral disc. Type D cases primarily opt for left O-ALIF. Besides the primary approach, other options can be considered based on a comprehensive analysis including the patient's gender, the presence of a fatty gap between the common/internal iliac veins and the intervertebral disc, the number of surgical segments, and the surgeon's experience. This classification system is simple, intuitive, and the primary approaches determined by this system are reliable in preventing injury to the common/internal iliac veins and the superior hypogastric plexus.

## Data Availability

The raw data supporting the conclusions of this article will be made available by the authors, without undue reservation.
